# Effects of Perceived Management Care and Job Embeddedness on Organizational Commitment Among Registered Clinical Nurses: A Latent Profile Analysis

**DOI:** 10.1097/jnr.0000000000000742

**Published:** 2026-05-08

**Authors:** Zhiqian GONG, Dan CHEN, Yi HUANG, Yuwei WU, Huihui JIN

**Affiliations:** 1 School of Nursing, Hunan Normal University, Changsha, China; 2 Kiang Wu Nursing College of Macau, Macau, China; 3 School of Nursing, The University of Auckland, Auckland, New Zealand

**Keywords:** organizational commitment, nurse, latent profile analysis, perceived management care, job embeddedness

## Abstract

**Background:**

Nurses are the backbone of the health care service team, and having a stable team of nurses is critical to effective team operations. Organizational commitment helps nurses remain in their current organization and facilitates the attainment of organizational goals. Understanding the organizational commitment of nurses and its influencing factors is necessary.

**Purpose:**

This study was conducted to identify the latent characteristics affecting the level of organizational commitment in nurses and explore their influencing factors to provide feasible recommendations for improving hospital nursing management measures.

**Methods:**

From November 2023 to February 2024, 1,037 nurses from hospitals in Beijing, Changsha (Hunan Province), and Jinhua (Zhejiang Province) participated in this study. The data were collected using a socio-demographic information questionnaire, Organizational Commitment Scale, Global Job Embeddedness Items, and Caring Assessment Tool-administration. Data analysis included latent profile analysis, χ^2^ test, analysis of variance, and multifactorial logistic regression.

**Results:**

The level of organizational commitment may be classified into four distinct profiles, namely Observers (*n*=95, 9.16%), Stabilizers (*n*=319, 30.76%), Aspirants (*n*=369, 35.58%), and Stalwarts (*n*=254, 24.49%). The results of this study showed that higher levels of perceived management care, job embeddedness, and salary satisfaction, earning a lower monthly income, longer years of service, having children, and being employed in a permanent position were positively associated with higher organizational commitment.

**Conclusion/Implication for Future Practice:**

The findings indicate that organizational commitment in nurses is affected by factors including self-perceived management care, job embeddedness, salary satisfaction, employment form, whether they have children, monthly income, and years of service. The results of this study provide evidence that hospital nursing managers may reference in enhancing measures to strengthen the organizational commitment of nurses, which may include improving humane management practices and the work environment as well as rationalizing income distribution and human resources allocation, thereby promoting nurses’ identification with organizational goals, increasing retention, and enhancing the quality of nursing services and patient satisfaction.

## Introduction

The shortage of nurses has been a major challenge to global health care services for a long time, with more than 9 million vacancies projected by 2030 (World Health Organization, 2020). A stable nursing team is essential to maintaining the welfare of nursing staff and contributing to stable health care system operations (Pressley & Garside, 2023; Wang et al, 2025). Organizational Commitment (OC) refers to the degree of emotional attachment an employee has to their organization, their acceptance of the organization’s goals and values, and their desire to remain with their organization (Meyer & Allen, 1991). According to Mowday et al. (1979), OC represents how employees identify with their organization and the degree to which they actively participate in its activities. Meyer and Allen (1991) proposed a three-component model to explain the psychological state of individual OC, consisting of affective commitment (AC), continuance commitment (CC), and normative commitment (NC). AC refers to an individual’s emotional attachment to their organization, motivated by identification with the organization’s goals; CC refers to the cost an individual perceives in leaving the organization, including economic loss and the difficulty of finding new employment; and NC refers to the individual’s feeling of obligation to continue working for the organization (Meyer & Allen, 1991). Studies have shown that enhancing nurses’ OC can increase their job satisfaction, work effort, and job performance; foster a professional mission; and promote better quality of care (AI-Haroon & AI-Qahtani, 2020; AI-Jabari & Ghazzawi, 2019). Lack of commitment exacerbates burnout, reducing quality of care and furthering the shortage of nurses (Mon et al., 2022). Therefore, examining the factors affecting OC in nurses is important. Currently, the focus of quantitative studies has been primarily on antecedent variables such as leadership style (Dossary, 2022), work climate (Fantahun et al., 2023), and family-work conflict (Song et al., 2024). Studies conducted in China have shown that psychological capital (Chang et al., 2023) and transformational leadership (Deng et al., 2023) are positive predictors of OC in nurses.

According to the person-environment fit theory (Cooman & Vleugels, 2022), when the needs, values, goals, abilities, and personality of an employee align with the demands, supplies, values, and culture of their organization, they can adapt well to their environment. This good fit enables them to better handle their job demands and better understand their organization’s goals and values (van Zyl et al., 2023). The person-environment fit mostly addresses an employee’s fit with their organization, job, colleagues, leaders, and required skills, so managerial support and employee’s job embeddedness (JE) may improve the fit between nurses and their organization and subsequently improve OC.

The theory of nurse-perceived management care was proposed by Duffy (2013) based on Watson’s caring theory, highlighting that the essence of care is human interaction centered on caring aimed at enhancing others’ physical and mental health and that humanistic care is a core component of nursing (Watson, 2008). Prior findings show that nursing managers who promote professional competence in their nursing teams, demonstrate a democratic leadership style, and support work-life balance are perceived by nurses as exhibiting caring behaviors toward them (Peng et al., 2015). The results of studies conducted in Egypt (Faramawy & Kader, 2021) and China (Yang & Sun, 2021) have confirmed that nurses’ perceptions of management concerns can enhance their OC.

JE, which represents the sum of all factors preventing an individual from leaving their organization, is a predictor of intention to stay in nursing (Crossley et al., 2007). This concept includes the three components of fit, links, and sacrifice, which respectively represent the degree to which a nurse’s capabilities meet job demands; the degree of connection between a nurse and their managers, colleagues, and patients; and the resources a nurse would lose by leaving their organization (Reitz & Anderson, 2011). Research has shown associations in nurses between stronger JE and, respectively, deeper connection with their organization, stronger sense of belonging, better work engagement, and better recognition of and alignment with organizational goals and values (Zhang et al., 2012).

Latent profile analysis (LPA), a statistical method that classifies individuals based on different response patterns on continuous manifest variables, may be used to help identify group heterogeneity (Wen et al., 2023). This person-centered approach can identify relationships or subgroups within a variable system at different levels to explore future trends or factors influencing each type of research object, focusing on the study of individual variability (Wen et al., 2023). The focus of most related studies to date has been on using scale scores to judge the current state of nurses’ OC. Thus, they have not adequately considered the characteristics of nurses with different OC scores. Therefore, in this study, the person-environment fit theory was adopted as the theoretical framework, and LPA was employed to identify latent profiles and the population distributions of different levels of OC. The aim was to assist nursing managers in predicting identification with organizational values and turnover intentions in nurses within their nursing teams. Furthermore, the findings of this study are intended to support the development of targeted interventions to enhance organizational commitment, stabilize the nursing workforce, and, ultimately, improve the quality of nursing care.

## Methods

### Design and Participants

A convenience sampling method was used from November 2023 to February 2024 to recruit nurses from five secondary and higher hospitals across Beijing, Changsha (Hunan Province), and Jinhua (Zhejiang Province). The inclusion criteria were: (1) registered clinical nurse with professional qualifications; (2) work experience of at least one year; (3) providing informed consent and agreement to participate. The exclusion criteria were nurses currently in training or internships. LPA requires a minimum sample size of 500 cases (Nylund-Gibson & Choi, 2018), thus a minimum of 500 nurses were targeted as participants in this study.

### Measures

#### Socio-demographic information questionnaire

Based on the literature, a general information survey with a total of 13 entries was developed. The data collected included age, gender, hospital ranking, educational level, years of work experience, employment type, professional title, specialist nurse status, whether they have children, evening and night shift frequency (average per month), average weekly working hours in the last month, monthly income, and income satisfaction. The income satisfaction was rated on a Likert five-point scale, with 1 representing “*very dissatisfied*” and 5 representing “*very satisfied*”.

#### Organizational Commitment Scale

The Organizational Commitment Scale, developed by Allen and Meyer (1990), was previously translated into Chinese and validated by Lin (2000). This scale includes three dimensions, namely affective commitment (6 items), continuance commitment (6 items), and normative commitment (6 items), with a total of 18 items. Scoring is based on a Likert five-point scale, with 1 representing “*strongly disagree*” and 5 representing “*strongly agree*,” and higher scores indicating a higher level of OC. The total possible scale score ranges from 18 to 90, and the Cronbach alpha coefficients for each scale dimension range from .70 to .88.

#### Global Job Embeddedness Items

The Global Job Embeddedness Items, developed by Crossley et al. (2007) and previously translated into Chinese and validated by Mei et al. (2014), is a unidimensional scale with seven items, including (1) I feel attached to the organization; (2) it is difficult for me to make a decision to leave the organization; (3) I like my current organization and will not leave; (4) I am tired of the organization; (5) I cannot leave the organization rashly; (6) leaving the organization is easy for me, and (7) I feel closely connected to the organization. Scale scoring is done using a Likert five-point scale, with “*strongly disagree*” representing a score of 1 and “*strongly agree*” representing a score of 5. Items 4 and 6 are reverse-scored, with 1 representing “*strongly agree*” and 5 representing “*strongly disagree.*” Higher scores indicate higher levels of job embeddedness. The Cronbach alpha for the Chinese version of the scale is .83.

#### Caring Assessment Tool-administration

The Caring Assessment Tool-administration, developed by Duffy (2013) and translated into Chinese and validated by Peng et al. (2020), includes three dimensions, namely collaborative decision-making (12 items), respect (14 items), and noncaring (10 items), with a total of 36 items. CAT-admin is scored using a Likert five-point scale, with 1 representing “*never*” and 5 representing “*always.*” The noncare dimension is reverse-scored, with 1 representing “*always*” and 5 representing “*never.*” Higher scores indicate greater perceived care from managers. The Chinese scale version has a Cronbach alpha of .97 and a content validity index of .89.

### Ethical Considerations

This study was approved by the ethics committee of Hunan Normal University Biomedical Research on November 24, 2023 (2023-627) and was conducted in line with the principles of informed consent and voluntary participation. The participants were informed in advance of the purpose and significance of this study as well as of the anonymity and confidentiality of their questionnaire responses. Completing and submitting the questionnaire online was considered as providing informed consent.

### Data Collection

The questionnaires were distributed using the online survey platform “Wenjuanxing” (https://www.wjx.cn/). The purpose, significance, and ethical principles of this study were attached to the first page of the questionnaire. A QR code link was sent to WeChat groups used by nurses after contacting and obtaining consent from nursing department directors or ward managers at the five participating hospitals. In terms of the settings used on the “Wenjuanxing” platform, all of the questionnaire questions were set as mandatory, and the device restriction setting prevented multiple responses to ensure data integrity. The participants were allowed to withdraw from the study at any time, and their responses and personal information were not recorded during questionnaire completion. Upon completion, the surveys were checked individually, with responses completed in <120 s or showing obvious homogeneity or regularity options excluded. A total of 1,096 questionnaires were distributed, and 1,037 valid responses were recovered, with an effective rate of 94.6%.

### Data Analysis

The study data were analyzed using IBM SPSS Statistics 25.0 (IBM Corp., Armonk, NY, USA). Categorical data were described using frequencies and proportions. Normally distributed quantitative data were described using means and standard deviations, while non-normally distributed data were described using medians and quartiles. χ^2^ tests were used to determine the significant variables associated with the nurses’ OC in different profiles, and analysis of variance (ANOVA) was employed for comparisons of perceived management care and job embeddedness among different OC profiles. Multifactorial logistic regression was used to analyze factors influencing OC in nurses of different profiles based on statistically significant variables from the univariate analysis. The significance level was set at α=.05 for all tests, and a two-sided *p*<.05 was considered statistically significant.

Mplus 8.3 (Muthén & Muthén, Los Angeles, CA, USA) was used to conduct the latent profile analysis of OC, with the following criteria used to assess model fit: Bayesian Information Criterion (BIC), Akaike Information Criterion (AIC), adjusted Bayesian Information Criterion (aBIC), entropy, LoMendell-Rubin Adjusted Likelihood Ratio Test (LMRT), and Bootstrapped Likelihood Ratio Test (BLRT). Lower BIC and AIC values indicate better fit; entropy measures the certainty of assigning subjects into k profiles, with values closer to 1 indicating more precise results and values above 0.8 considered acceptable. Significant *p* values in LMRT and BLRT suggest models with k profiles to be superior to those with (k−1) profiles.

## Results

### Socio-Demographic Information Analysis

Of the 1,037 participants, 87.9% worked in tertiary hospitals, and the gender ratio was ~1:21 (male:female). The age range was from 20 to 40, with 22.1% at 20–25, 25.7% at 26–30, and 26.5% at 31–35 years old. In terms of education, 16.6% held an associate degree, 80.5% held a bachelor’s degree, and the remainder held a master’s degree or above. One-third (32.7%) had worked for <5 years, 25.7% had worked for 6–10 years, and the remainder had worked for over 10 years. Nearly two-thirds (62.2%) were permanently employed, while the others were employed on a contract or through a personnel agency. A minority (12.4%) were specialist nurses. In terms of professional title, 22.5% were nurses, 31.4% were nurse practitioners, and 37.8% were nurse supervisors, with the remainder serving in deputy head nurse or higher positions. Over half (58.6%) had children. In terms of average monthly evening and night shift frequency, 21.9% had no evening and night shifts, 37.4% had 5, and others had more than 6. Most (87.1%) worked 40 or more hours on average per week. In terms of monthly income, 26.5% earned <5,000 yuan, 55.2% between 5,000 and 8,000 yuan, and only 4.1% more than 10,000 yuan. One-third (32.7%) were satisfied with their income, while 20.3% were unsatisfied.

### Latent Profile Analysis of Organizational Commitment

The 18 items of the Organizational Commitment Scale were used as manifest variables in the LPA, with profiles ranging from 1 to 6. The results indicated that as the number of profiles increased, the AIC, BIC, and SSA-BIC values decreased, and that entropy initially increased and then decreased, suggesting a more precise classification as entropy approaches 1. When selecting five profiles, the entropy was 0.929, and the LMRT did not show statistical significance, indicating a less-optimal model fit compared with four profiles, where the highest entropy was 0.958, and both LMRT and BLRT were statistically significant (see Table 1 for details).

**Table 1:** Model Fitting Indexes for the Latent Profile Analysis of Organizational Commitment

No. Classes	AIC	BIC	SSA-BIC	RFSC (%)	Entropy	*p* (LMRT)	*p* (BLRT)
1	54961.24	55139.23	55024.89	—	—	—	—
2	48066.30	48338.22	48163.54	46.3	0.948	<.001	<.001
3	45842.59	46208.45	45973.42	10.6	0.951	<.001	<.001
4	44098.35	44558.15	44262.77	9.2	0.958	<.001	<.001
5	43568.72	44122.45	43766.73	9.0	0.929	.337	<.001
6	43123.93	43771.61	43355.54	7.8	0.928	.632	<.001

*Note*. AIC = Akaike information criterion; BIC = Bayesian information criterion; SSA-BIC = sample-size adjusted Bayesian information criterion; RFSC=relative frequency of smallest class; LMRT = Lo–Mendell–Rubin adjusted likelihood ratio test; BLRT = bootstrapped likelihood ratio test.

**Figure 1:**
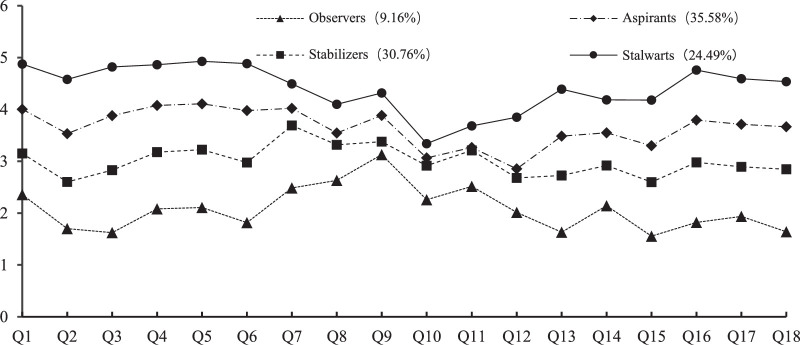
The Mean Scores for Each Organizational Commitment Item in the Four Profiles

Based on the LPA results, four latent profiles were identified for OC across the 18 manifest indicators, as shown in Figure 1. Each latent profile was named according to the scores of each entry. Profile 1 was characterized as having the lowest overall level of OC, with the level of CC higher than that of either AC or NC. This pattern indicates that Profile 1 is a subgroup typified by uncommitted or CC-dominant nurses with an OC based on utilitarian considerations related to the feasibility of leaving (e.g., compensation, new employment opportunities), and low levels of emotional dependence and sense of responsibility. Profile 1 nurses maintain a wait-and-see attitude toward their organization and await the emergence of better job opportunities. Based on the above description, this subgroup was named “Observers” in this study. Profile 2 is characterized by CC-dominant individuals and is associated with a higher OC level and greater balance among the three dimensions than the Observers. Profile 2 individuals have a stable attitude toward retention within their organization. Based on the above description, this subgroup was named “Stabilizers” in this study. Profile 3 is co-dominated by AC and NC, with both levels higher than CC. Compared with the previous two groups, they exhibit higher levels of emotional dependence and moral obligation toward their organization. Also, rather than emphasizing the value of their job opportunity and compensation, they place greater value on achieving organizational goals. Based on the above description, this subgroup was named “Aspirants” in this study. Profile 4 was associated with the highest OC level and the highest AC, making it the “fully committed” or AC-dominant group. The commitment of nurses in this subgroup to their organization is based primarily on their strong affective attachment and sense of responsibility. They perceive work as a personal ideal and achievement and demonstrate a strong commitment to staying with their organization. Based on the above description, this subgroup was named “Stalwarts” in this study.

### Analysis of the Organizational Commitment of the Profile Subgroups

The results of the univariate analysis identified significant differences among the OC profile subgroups in terms of hospital level, age, years of work, employment type, specialist nurse status, professional title, having children, monthly night shifts, weekly working hours, monthly income, and income satisfaction (*p*<.05). Moreover, perceived management care and job embeddedness varied significantly across the profiles (*p*<.05), as detailed in Tables 2 and 3. The results of the post hoc comparison showing that the group with higher OC levels had stronger perceived management care and job embeddedness are shown in Table 3.

**Table 2:** Univariate Analysis of Socio-Demographic Information, by OC Profile (*N*=1,037)

Variable	Observers (*n*=95)	Stabilizers (*n*=319)	Aspirants (*n*=369)	Stalwarts (*n*=254)	χ^2^	*p*
Hospital ranking					16.17	.001
Tertiary	91 (95.8)	292 (91.5)	318 (86.2)	211 (83.1)		
Secondary	4 (4.2)	27 (8.5)	51 (13.8)	43 (16.9)		
Sex					6.20	.102
Male	7 (7.4)	19 (6.0)	16 (4.3)	6 (2.4)		
Female	88 (92.6)	300 (94.0)	353 (95.7)	248 (97.6)		
Age					95.89	<.001
20–25	30 (31.6)	89 (27.9)	80 (21.7)	30 (11.8)		
26–30	27 (28.4)	112 (35.1)	83 (22.5)	44 (17.3)		
31–35	21 (22.1)	73 (22.9)	112 (30.4)	69 (27.2)		
36–40	17 (17.9)	45 (14.1)	94 (25.5)	111 (43.7)		
Educational level					5.45	.488
Associate degree	14 (14.7)	61 (19.1)	58 (15.7)	39 (15.4)		
Bachelor’s degree	79 (83.2)	248 (77.7)	297 (80.5)	211 (83.1)		
Master’s degree or higher	2 (2.1)	10 (3.1)	14 (3.8)	4 (1.6)		
Years of work experience					95.62	<.001
<3	22 (23.2)	74 (23.2)	75 (20.3)	30 (11.8)		
3–5	17 (17.9)	61 (19.1)	43 (11.7)	17 (6.7)		
6–10	27 (28.4)	98 (30.7)	93 (25.2)	49 (19.3)		
11–15	20 (21.1)	60 (18.8)	89 (24.1)	75 (29.5)		
16–20	9 (9.5)	26 (8.2)	69 (18.7)	83 (32.7)		
Employment type					100.99	<.001
Permanent employment	43 (45.3)	220 (69.0)	244 (66.1)	138 (54.3)		
Contract-based	24 (25.3)	83 (26.0)	120 (32.5)	112 (44.1)		
Personnel agency	28 (29.5)	16 (5.0)	5 (1.4)	4 (1.6)		
Specialist nurse status					24.67	<.001
Yes	6 (6.3)	21 (6.6)	54 (14.6)	48 (18.9)		
No	89 (93.7)	298 (93.4)	315 (85.4)	206 (81.1)		
Professional title					56.80	<.001
Nurse	22 (23.2)	88 (27.6)	78 (21.1)	45 (17.7)		
Nurse practitioner	39 (41.1)	118 (37.0)	112 (30.4)	57 (22.4)		
Nurse supervisor	32 (33.7)	101 (31.7)	148 (40.1)	111 (43.7)		
Deputy head nurse and above	2 (2.1)	12 (3.8)	31 (8.4)	41 (16.1)		
Have children?					65.66	<.001
Yes	40 (42.1)	147 (46.1)	227 (61.5)	194 (76.4)		
No	55 (57.9)	172 (53.9)	142 (38.5)	60 (23.6)		
Evening and night shift frequency (monthly average)					131.98	<.001
0	6 (6.3)	38 (11.9)	74 (20.1)	109 (42.9)		
≤ 5	33 (34.7)	108 (33.9)	154 (41.7)	93 (36.6)		
6–10	45 (47.4)	147 (46.1)	125 (33.9)	47 (18.5)		
≥ 11	11 (11.6)	26 (8.2)	16 (4.3)	5 (2.0)		
Average weekly working hours					49.80	<.001
<40	9 (9.5)	19 (6.0)	53 (14.4)	53 (20.9)		
40	24 (25.3)	89 (27.9)	115 (31.2)	97 (38.2)		
>40	62 (65.3)	211 (66.1)	201 (54.5)	104 (40.9)		
Monthly income (CNY)					21.88	.009
<5,000	25 (26.3)	84 (26.3)	93 (25.2)	73 (28.7)		
5,000–8,000	55 (57.9)	197 (61.8)	193 (52.3)	127 (50.0)		
8,001–10,000	11 (11.6)	25 (7.8)	67 (18.2)	44 (17.3)		
>10,000	4 (4.2)	13 (4.1)	16 (4.3)	10 (3.9)		
Income satisfaction					318.89	<.001
Very satisfied	38 (40.0)	53 (16.6)	22 (6.0)	6 (2.4)		
Somewhat satisfied	28 (29.5)	97 (30.4)	75 (20.3)	20 (7.9)		
Neutral	27 (28.4)	154 (48.3)	204 (55.3)	102 (40.2)		
Somewhat dissatisfied	2 (2.1)	12 (3.8)	63 (17.1)	88 (34.6)		
Very dissatisfied	0 (0.0)	3 (0.9)	5 (1.4)	38 (15.0)		

*Note.* CNY=Chinese yuan.

**Table 3:** Comparison of Perceived Management Care and Job Embeddedness Across Different Organizational Commitment Profiles (*N*=1,037)

Variable	Total	① Observers (*n*=95)	② Stabilizers (*n*=319)	③ Aspirants (*n*=369)	④ Stalwarts (*n*=254)	*F*	*p*	SNK
Perceived management care	139.88±23.95	118.68±21.65	127.25±21.16	142.84±19.96	159.35±16.41	165.696	<.001	④>③>②>①
Collaborative decision-making	44.86±9.41	35.75±8.28	39.95±7.78	46.23±7.86	52.44±6.98	174.950	<.001	④>③>②>①
Respect	55.68±10.49	45.92±10.23	50.10±9.51	57.39±8.48	63.84±6.85	165.538	<.001	④>③>②>①
Noncaring	39.34±7.71	37.02±6.25	37.20±7.06	39.21±8.36	43.07±6.56	33.786	<.001	④>③>②,④>③>①
Job embeddedness	22.97±3.31	18.94±2.77	21.30±2.42	23.85±2.84	25.29±2.62	193.212	<.001	④>③>②>①

*Note.* SNK = Student–Newman–Keuls test.

### Multifactorial Logistic Regression Analysis of Organizational Commitment Latent Profiles

Next, a multinomial logistic regression analysis was performed to validate the factors of influence on OC across the four profiles, with Observers serving as the reference group. As shown in Table 4. Participants with monthly incomes under 8,000 yuan were significantly more likely to be grouped into Aspirants and Stalwarts than into the Observers subgroup. Also, those with children were significantly more likely to be in the Stalwarts subgroup. Furthermore, Observers were more likely than Stalwarts to have 3–10 years rather than 16–20 years of working experience. Compared with Observers, all of the other subgroups had higher levels of perceived management care, job embeddedness, and income satisfaction. Those participants with permanent or contract-based employment were more likely to be in the higher OC profile level subgroups.

**Table 4:** Results of Multifactorial Logistic Regression Analysis (*N*=1,037)

Variable	Stabilizer			Aspirant			Stalwart		
	β	*p*	*OR* [95% CI]	β	*p*	*OR* [95% CI]	β	*p*	*OR* [95% CI]
Perceived management care	0.012	.088	1.012 [0.999, 1.026]	0.042	<.001	1.043 [1.027, 1.059]	0.082	<.001	1.086 [1.066, 1.105]
Job embeddedness	0.349	<.001	1.418 [1.272, 1.582]	0.683	<.001	1.979 [1.744, 2.247]	0.833	<.001	2.300 [1.996, 2.649]
Income satisfaction	0.799	<.001	2.223 [1.561, 3.164]	1.317	<.001	3.733 [2.502, 5.569]	2.153	<.001	8.609 [5.459, 13.577]
Employment type									
Permanent employment	2.489	<.001	12.052 [5.154, 28.186]	3.645	<.001	38.276 [10.662, 137.415]	2.531	.002	12.570 [2.592, 60.968]
Contract-based	2.139	<.001	8.494 [3.371, 21.399]	3.622	<.001	37.421 [9.792, 143.012]	2.892	<.001	18.035 [3.542, 91.821]
Personnel agency			1.000			1.000			1.000
Have children?									
Yes	0.562	.173	1.754 [0.782, 3.932]	0.872	.058	2.392 [0.971, 5.895]	1.407	.008	4.085 [1.445, 11.550]
No			1.000			1.000			1.000
Monthly income									
<5,000	1.200	.096	3.319 [0.809, 13.623]	1.997	.017	6.371 [1.427, 38.057]	3.286	.001	26.725 [4.145, 172.308]
5,000–8,000	0.856	.207	2.355 [0.623, 8.902]	1.416	.074	4.120 [0.873, 19.451]	2.420	.007	11.250 [1.927, 65.672]
8,001–10,000	-0.497	.516	0.516 [0.136, 2.719]	0.395	.649	1.484 [0.271, 8.117]	0.749	.443	2.115 [0.312, 14.347]
>10,000			1.000			1.000			1.000
Years of work experience									
<3	0.756	.271	2.129 [0.555, 8.165]	−0.039	.959	0.962 [0.224, 4.140]	−1.143	.167	0.319 [0.063, 1.615]
3–5	0.679	.296	1.973 [0.551, 7.063]	−0.480	.506	0.619 [0.151, 2.543]	−1.885	.021	0.152 [0.030, 0.757]
6–10	0.617	.287	1.854 [0.595, 5.775]	−0.343	.580	0.710 [0.211, 2.385]	−1.378	.039	0.252 [0.068, 0.933]
11–15	0.173	.748	1.189 [0.413, 3.419]	−0.369	.520	0.691 [0.225, 2.126]	−0.784	.200	0.457 [0.137, 1.516]
16–20			1.000			1.000			1.000

*Note.* “Stabilizers,” “Aspirants,” and “Stalwarts” are all based on “Observers” as the reference group.

## Discussion

In this study, LPA was applied to comprehensively analyze the OC characteristics of the participants, which were grouped into four profile subgroups marked by a steadily increasing level of OC, that is, 9.16% for Observers, 30.76% for Stabilizers, 35.58% for Aspirants, and 24.49% for Stalwarts. The finding that Aspirants were associated with the most and Observers associated with the least number of participants indicates a medium level of OC in this sample, which aligns with the findings of Cao et al. (2019) and Tang et al. (2022) that most Chinese nurses exhibit a strong desire to remain within their current organizations. Moreover, the factors influencing different profiles and the correlations among perceived management care, job embeddedness, and OC were explored in this study. The results indicate the main factors forming the four profiles are: perceived management care, job embeddedness, income satisfaction, employment type, having/not having children, monthly income, and years of work experience.

### Analysis of Profile Characteristics

The CC score was highest in the Observers and Stabilizers subgroups. Prior research has shown that CC reflects employee attachment based on instrumental considerations related to the perceived lack of other employment alternatives and leaving-related sacrifices (Stanley et al., 2013). Moreover, CC is a form of commitment that is externally regulated and viewed as non–self-decided in theories of motivation (Stanley et al., 2013). In this study, both subgroups reported the lowest scores for item 12 (“I believe I have too few options to consider leaving this organization”). The highest CC score for the Observers subgroup was item 9 (“Too much of my life would be disrupted if I leave my organization”), while the highest for the Stabilizers subgroup was item 7 (“Right now, staying with my job at this organization is a matter of necessity as much as desire”). This suggests a low perception of lacking employment options, potentially due to the participants having a relatively high average level of education, leading to self-confidence in their job competence and social competitiveness. However, the participants generally treasured the livelihood security (e.g., salary, benefits) provided by their organization and perceived the loss of income that would result from leaving, encouraging them to remain. This may relate to the traditional Chinese mindset of living in peace and contentment, which fosters the aspiration to hold a stable job and a source of income to maintain a peaceful pace of life (Cruz, 2023). Therefore, compared with nurses of other nationalities, nurses in China may be more resistant to changing the pace and state of their lives by taking another job that may not be any better than the one they have at the moment. Coping with a new organization is challenging and complex. Participants in the Stabilizers subgroup also reflected higher overall OC levels because of their higher AC and NC levels than Observers. Therefore, it seems important to ensure nurses are provided with reasonable, fair salaries and chances to develop specific job skills. For nurses with low levels of commitment, managers should focus on fostering emotional attachment and a sense of moral obligation to the organization. This can help nurses shift from passive employment driven by utilitarianism to active retention based on shared values and responsibility, leading to greater commitment.

In this study, the Aspirants subgroup was the largest, accounting for 35.58% of all participants. This subgroup had the highest average scores for AC and NC. Similarly, among the three dimensions, AC was highest in the Stalwarts subgroup (24.49% of all participants), followed by NC. The Aspirants and Stalwarts subgroups had higher scores for item 5 (“I feel like ‘part of the family’ at my organization”) than the other two subgroups, and reflect a strong sense of belonging and emotional identification with their organization. Consistent with Roth et al. (2022), nursing is a highly professional occupation characterized by helping others that holds wide public respect. Many nurses take pride in their profession and seek to enhance public recognition for their profession through their individual efforts, viewing nursing as a personal mission and choosing to remain. In addition, as China’s strong collectivist culture values relationships with colleagues and leaders and organizational loyalty (Gong et al., 2021), nurses see achieving organizational goals as a duty and obligation.

In the four profiles, **c**ompared with the other two dimensions, CC scores appeared more clustered in the profile figure, indicating smaller differences across the four profiles. Compared with Observers and Stabilizers, the Aspirants and Stalwarts subgroups had significantly higher scores for AC and NC, suggesting emotional attachment and sense of responsibility to the organization varies considerably among nurses. High OC levels relate to organizational retention and effectively explain differences in job satisfaction and patient safety culture (Gider et al., 2019). If nurses exhibit positive CC due to perceiving a lack of alternative employment opportunities, whether this perception relates to their social environment (e.g., society’s overall difficulty in finding a job) or personal reasons (e.g., a perceived lack of competitive employment advantage), they may feel involuntarily stuck in their current organization. Thus, organizations facing financial problems that at least temporarily reduce the material conditions of their nursing staff may see nurses who do not identify with the organization’s goals leave rather than share the organization’s difficulties. Therefore, nurses who identify as “wanting but being unable to leave” can negatively influence organizational stability. The CC-dominant commitment may not be conducive to the long-term stability and healthy development of either the individual or their organization. It is particularly important to enhance the desire of nurses to contribute to the achievement of their organization’s goals. Therefore, managers should focus on educating nurses on professional identity, helping them discover their personal mission and sense of professional purpose.

In terms of socio-demographics, Stalwarts were older (ages 35–40), had a longer tenure, had higher professional titles, were more likely to have children, worked fewer evening and night shifts per month, and had higher monthly income satisfaction than Observers. These findings are consistent with Mon et al. (2022) and Labrague et al. (2018), who reported age and work experience to be associated with higher OC in nurses. The results of related studies conducted in China indicate that income satisfaction impacts CC in nurses (Ye, 2021). Also, nurses who are older with more work experience have stronger competencies and confidence and feel more empowered by their managers, which enhances their perceived control over their work and strengthens their NC level (Fragkos et al., 2020). In addition, nurses with children tend to adopt a family-centered orientation, prioritizing job stability to manage caregiving responsibilities and associated financial demands while striving to balance multiple roles (Carve & Candel, 2008; Shujaat et al., 2019). Conversely, younger nurses are more open to new challenges and seek jobs with better career prospects (Chen et al., 2024). Less constrained by childcare and family responsibilities, younger nurses are freer, which makes changing jobs and relocating relatively easy. Therefore, they are more likely to leave if they find positions with better pay, more continuing education opportunities, and better career prospects (Cai et al., 2023). This suggests managers should pay close attention to identifying the Observers among their staff and determining their retention intentions, as well as to helping younger nurses raise their AC and NC levels. Simultaneously, attention should be given to older nurses’ balance between family and work to prevent family stress from affecting their quality of care (Yang et al., 2022).

### Analysis of Factors Influencing OC in the Subgroups

#### Perceived management care

The multifactorial logistic regression analysis results show that the participants with higher levels of perceived management care were more likely to exhibit higher OC and be in the Aspirants and Stalwarts subgroups. Furthermore, the results of the ANOVA pairwise comparison suggest that high OC is associated with higher self-perceived management care. A qualitative study conducted in Iran revealed nursing manager leadership style to be a significant factor influencing OC in nurses, with the management-led empowerment of nurses helping make nurses feel more valued by their organization and more conscious of their professional responsibilities (Sepahvand et al., 2019). Person-environment fit theory suggests that the fit between individuals and their managers can promote inclusiveness and certainty at work. Managers often have a deeper understanding than nurses of organizational values and goals, and thus can influence nurses’ knowledge of these values and goals during daily communications (Vianen, 2018). Regular communication, care, and encouragement; ensuring a safe working environment; treating nurses with respect and appreciation; and supporting their professional development can all help promote nurses' understanding of the way managers work, leading to improved fit with managers and increased emotional commitment, which can enhance the work attitudes and performance of nurses. These findings underscore the importance of training hospital management in humanistic and caring management practices.

#### Job embeddedness

The participants with higher JE levels clustered in the Stabilizers, Aspirants, and Stalwarts subgroups. Pairwise comparisons of the ANOVA analysis results show that the participants with high levels of OC also exhibited higher levels of JE. This is similar to a study conducted in Thailand that reported higher levels of JE to be a predictor of intention to stay in nursing (Dechawatanapaisal, 2018). Person-environment fit theory confirms that work relationships, support, and compensation benefits are crucial in aligning individuals with their environments. Despite the lack of explicit correlation between individual and organizational values, individuals can, through adaptation and learning, achieve value congruence with their organization to ensure they work toward organizational goals (Vianen, 2018). According to the three elements of JE, nurses who feel capable of achieving organizational goals and whose skills are well-matched to their job requirements tend to exhibit more positive work attitudes and a stronger sense of responsibility, thereby enhancing their NC (Gutierrez et al., 2012). Close relationships with organizational members were also found to help the participants build tight connections with the organization. Communication and exchanges with others can lead to new understandings of the organization and reinforce previous value alignments, enhancing value congruence and AC (Yan et al., 2023). When these elements became more substantial and diverse, nurses are less likely to leave, thereby reinforcing their CC. This analysis suggests managers should address the unmet work-environment needs and challenges of their nurses by adjusting and improving management systems and rationalizing human resource allocations to enhance their JE and organizational commitment.

#### Employment form and years of service

According to the results of the multifactorial logistic regression, nurses in permanent positions may be expected to be more likely to be categorized into the Stabilizers, Aspirants, or Stalwarts subgroups, and those with children and longer tenure are more likely to fit into the Stalwarts subgroup. In China, having a permanent job in the public sector is a long-term and stable form of employment, with nurses not needing to consider seeking new employment after contract expiration and typically benefitting from better salaries and perks. Therefore, hospitals often use permanent positions as a strategy to attract talent. However, in recent years, due to the limited number of permanent positions and the increasing demand for nursing staff, hospitals have increasingly employed a mixed personnel system comprised of permanent and nonpermanent staff (Zhou et al., 2020), leading to disparities in compensation for the same work. This situation often results in lower NC levels among nonpermanent nurses. Managers are advised to pay attention to the compensation of nonpermanent nurses, respond to national public sector management reforms, and reduce the pay gap between permanent and nonpermanent nursing staff.

Longer tenure implies richer work experience. According to Meyer and Allen (1991), as individuals progress in their tenure within an organization, they develop emotional attachments, believing they have an indispensable responsibility to help their organization achieve success. This self-enhancement leads to higher levels of NC, manifested in positive work attitudes, a heightened sense of personal achievement, and a desire to remain (Cherian et al., 2018). Nurses with long tenures are more familiar with their work environment, possess a deeper understanding of their organization’s goals and values, and have a higher level of AC. When it comes to leaving the organization, although their previous work experience may be sufficient to manage new roles, becoming familiar with a new environment would still require considerable effort. Thus, they demonstrated high levels of CC and showed low intention to pursue alternative employment opportunities (Sepahvand et al., 2019).

#### Income satisfaction and monthly income

The participants with a higher level of satisfaction with their income were concentrated in the Stabilizers, Aspirants, and Stalwarts subgroups, and those with lower monthly incomes were concentrated in the Aspirants and Stalwarts subgroups. Thus, while having a low-income level did not necessarily correlate with low OC, income satisfaction was found to directly influence OC. One possible explanation is that high-income levels are often associated with more work, greater responsibilities, and greater job stress (Labrague et al., 2018). Conversely, nurses with lower incomes may work in relatively less-demanding positions that carry lighter burdens, with their existing income sufficient for their living needs. Hence, lower-earning nurses are not compelled to seek higher-paying, more strenuous jobs, which results in higher AC toward their organization. However, income satisfaction reflects attitude toward earnings and evaluates whether their compensation matches the work within their responsibility scope and whether additional work is adequately rewarded. Managers should pay particular attention to individuals with high job stress who receive salaries near the societal average and report low income satisfaction. Globally, due to the rising trend in nursing education, high clinical workloads coupled with rising costs of living make nurses generally dissatisfied with their income as being not commensurate with their work contributions (Tamata & Mohammadnezhad, 2023). The findings of numerous studies indicate nurses perceive their workload far exceeds their compensation level, which can reduce their CC (Sepahvand et al., 2019; Ye, 2021). Despite being dissatisfied with the gap between work burden and salary expectations, many nurses still regard nursing as an important source of income. This highlights the importance of nursing managers considering fair and reasonable salaries and benefits as crucial in retaining nursing talent and stabilizing the nursing team. Managers should establish a clear salary distribution system, offer financial rewards for performing additional tasks, implement a system of evening and night shift subsidies, and ensure nurses do not bear the cost of continued training and professional development. Also, providing on-site welfare facilities such as fitness and recreational services may be beneficial in reducing the need for nurses to seek and pay for such services elsewhere.

### Implications

Firstly, four different OC profiles for Chinese nurses were identified using latent profile analysis. These were Observers (9.16%), Stabilizers (30.76%), Aspirants (35.58%), and Stalwarts (24.49%). The characteristics of each profile was explored to help managers better predict the OC of their nurses and take individualized measures to enhance the OC of uncommitted and CC-dominant nurses. Secondly, guided by person-environment fit theory, the relationships between OC profile and perceived management care and job embeddedness were investigated to further elucidate the factors influencing OC in nurses. This may help managers develop and apply effective caring strategies and promote job embeddedness to strengthen nurse commitment, reduce turnover, and stabilize the nursing team. OC in the Observers subgroup was low and characterized by CC-dominant commitment. Although Observers encompassed the lowest number of participants, the needs of this subgroup should be considered and addressed by managers. Targeted measures should be taken to enhance the overall commitment of the entire nurse team. Organizational and leadership support are the two most important aspects involved in enhancing commitment. The results of this study can help managers infer OC level in nurses by assessing their general situation, perceptions regarding management care, and level of organizational embeddedness. Based on their assessment, managers may then work to enhance AC and NC levels in CC-dominated nurses and strengthen the comprehensiveness and efficiency of management work.

### Limitations

This study is affected by several limitations. Firstly, only 1,037 nurses were surveyed in three provinces in China. Thus, the findings may not be representative of nurses nationwide. Secondly, the reliance in this study on self-reported data may introduce respondent biases that conceal the actual situation. Also, the cross-sectional survey method used may overlook certain factors that may influence OC level as nurses gain work experience. Lastly, as this study was conducted in the cultural context of China, the findings may be influenced by Chinese cultural norms and thus not be generalizable to other cultural contexts.

### Conclusions

In this study, latent profile analysis was used to identify four OC profiles related to nurses. The Aspirants subgroup contained the most participants. Older nurses who were more satisfied with their salary were more likely to be categorized into high-OC-level profile subgroups. Self-perceived management care, job embeddedness, income satisfaction, employment status, and tenure were found to significantly influence OC level. Based on the findings, managers should first strengthen their humanistic care-related training, ensure salaries are fairly distributed, provide nonpermanent staff with opportunities for professional development, and offer continuing education opportunities to nurses with shorter tenures. This may be expected to enhance job embeddedness, optimize the nursing team, improve working conditions, and raise commitment levels, thereby improving nursing quality. Future research may employ a mix of subjective and objective methods to study the trajectory of OC among senior nurses as their tenure lengthens and explore factors that motivate them to remain with their organization, guiding strategies to enhance OC and retention intention among junior nurses and managers.
